# Predicting real world spatial disorientation in Alzheimer’s disease patients using virtual reality navigation tests

**DOI:** 10.1038/s41598-022-17634-w

**Published:** 2022-08-04

**Authors:** Vaisakh Puthusseryppady, Sol Morrissey, Hugo Spiers, Martyn Patel, Michael Hornberger

**Affiliations:** 1 Norwich Medical School, 2.04 Bob Champion Research and Education Building, University of East Anglia, Norwich, NR4 7TJ UK; 2 Department of Neurobiology and Behaviour, University of California Irvine, Irvine, 92617 USA; 3 Division of Psychology and Language Sciences, Department of Experimental Psychology, Institute of Behavioural Neuroscience, University College London, London, WC1H 0AP UK; 4 Norfolk and Norwich University Hospitals National Health Service (NHS) Foundation Trust, Colney Lane, Norwich, NR4 7UY UK

**Keywords:** Cognitive ageing, Cognitive neuroscience, Diseases of the nervous system, Alzheimer's disease, Human behaviour

## Abstract

Spatial navigation impairments in Alzheimer’s disease (AD) have been suggested to underlie patients experiencing spatial disorientation. Though many studies have highlighted navigation impairments for AD patients in virtual reality (VR) environments, the extent to which these impairments predict a patient’s risk for spatial disorientation in the real world is still poorly understood. The aims of this study were to (a) investigate the spatial navigation abilities of AD patients in VR environments as well as in a real world community setting and (b) explore whether we could predict patients at a high risk for spatial disorientation in the community based on their VR navigation. Sixteen community-dwelling AD patients and 21 age/gender matched controls were assessed on their egocentric and allocentric navigation abilities in VR environments using the Virtual Supermarket Test (VST) and Sea Hero Quest (SHQ) as well as in the community using the Detour Navigation Test (DNT). When compared to controls, AD patients exhibited impairments on the VST, SHQ, and DNT. For patients, only SHQ wayfinding distance and wayfinding duration significantly predicted composite disorientation score on the DNT (β = 0.422, *p* = 0.034, R^2^ = 0.299 and β = 0.357, *p* = 0.046, R^2^ = 0.27 respectively). However, these same VR measures could not reliably predict which patients were at highest risk of spatial disorientation in the community (*p* > 0.1). Future studies should focus on developing VR-based tests which can predict AD patients at high risk of getting spatially disorientated in the real world.

## Introduction

Spatial navigation is one of the earliest cognitive domains to be impaired in patients with Alzheimer’s disease (AD), resulting in affected individuals experiencing spatial disorientation^1^. Spatial disorientation refers to instances where a patient is unaware of their whereabouts and unable to navigate to an intended location^2^. Indeed, this behavioural symptom causes patients to make navigation errors whilst in the community and subsequently can result in them getting lost from both unfamiliar and familiar environments^3^. Previous studies have reported that up to 70% of patients experience at least one getting lost episode over the course of the disease, with some even at risk for experiencing multiple episodes^4–7^. It has also been reported that up to 40,000 patients get lost in the community for the first time every year in the UK, and these incidence rates are likely to grow in the coming years with the projected increase in the dementia patient population seen globally^4,8^.

Being a prevalent problem worldwide, AD patients getting lost in the community is associated with a wide range of negative consequences. For patients, these episodes can increase their chances of being admitted to a care home by seven times, cause them to feel a decreased sense of autonomy, as well as increase their chances of sustaining injuries and in the worst cases, even potential death^6,9^. Moreover, having to routinely deal with patients getting lost in the community can lead to increased carer burden and distress as well as trigger the increasing involvement of law enforcement groups and community search resources^10–13^. Hence overall, AD patients getting lost in the community not only affects the patients themselves, but also their carers, and the wider community in which they live in.

Considering the large scope of the problem, there has been increasing research interest into understanding why spatial disorientation, and subsequently the getting lost episodes, occur for AD patients. From a neuroscience perspective, spatial disorientation has been suggested to occur as a result of impairments in spatial navigation, and therefore studies have in large investigated how spatial navigation is affected in AD patients. To explore this concept, researchers have in recent years taken advantage of the advent of virtual reality (VR) environments, which have been used to test the spatial navigation abilities of AD patients as well as its underlying neural correlates. The results of these VR navigation studies suggest that patients exhibit impairments in the ability to use both egocentric (eye-/head/body-based) and allocentric (map-based) navigation strategies, which is associated with pathology related deficits seen respectively in the parietal and medial temporal lobe structures. Further, impairments in the ability to switch between the two navigation strategies have also been reported in patients^1,14^.

Although the VR navigation studies have been integral in furthering understanding of how spatial navigation is affected in AD, a critical research gap associated with these studies is that they have not in large related the measured VR navigation impairments in patients to them experiencing spatial disorientation in the community. Some studies have related the navigation performance of AD patients in VR environments to their performance in real world analogues of the same environments, however the latter environments are often unfamiliar and controlled in design^15–18^. As a result, these types of environments often do not capture the complexity of familiar locations in the community, which is where AD patients most commonly experience spatial disorientation^10,19^. Furthermore, most VR navigation studies have utilised non-immersive, desktop environments that prevent participants from using body-based cues (a key source of input for navigation), and VR environments have also been shown to induce cyber sickness in AD patients^20^. These factors could all indeed result in VR environments not accurately simulating real world situations where spatial disorientation may occur for patients. Therefore at present, the extent to which spatial navigation impairments measured using VR predicts patients’ risk for spatial disorientation in the community is still poorly understood. As spatial disorientation is highly unpredictable in its onset and associated with potentially fatal consequences, identifying patients at high risk for spatial disorientation is of clear importance due to not only its implications in preventing this subgroup of individuals from getting lost in the future but also due to social implications, with regards to encouraging those not at a high risk to maintain their autonomy in the community for as long as possible.

In this study, we aim to address this research gap by first systematically investigating how AD patients navigate in VR settings followed by how they navigate in a familiar community setting, in a situation where spatial disorientation is likely to occur. We then relate findings from both tests to explore whether we can predict which patients are at a high risk for spatial disorientation in the community based on their performance in the VR navigation tests. We hypothesise that patients will exhibit impaired performance on both the VR and community navigation tests, as AD patients are widely reported to be impaired in navigating through both VR and RW environments^14^. We also hypothesise that patients who perform relatively worse on the egocentric orientation components of the VR tests will in turn be the ones that exhibit more spatial disorientation in the community test. This is because findings from previous studies have suggested that AD patients rely and use more of an egocentric strategy to navigate in the real world, potentially as a means to compensate for early impairments to their allocentric navigation abilities^17,21,22^. Hence, we hypothesise that those with relatively weaker egocentric orientation abilities will be less able to use this strategy to aid their navigation, and hence be at higher risk for experiencing spatial disorientation. It is envisioned that such a finding would enhance the real-world applications of the VR navigation tests that we use, towards risk stratification of a patients’ propensity for spatial disorientation in the community.

## Methods

### Participant recruitment

A total of 16 community residing AD patients as well as 21 age and gender-matched healthy controls were recruited for this study (see Supplementary Material for details on recruitment). All patients were clinically diagnosed with AD using the National Institute of Neurological and Communicative Disorders and Stroke and the Alzheimer’s Disease and Related Disorders Association (NINCDS/ADRDA) diagnostic criteria^23^.

All recruited participants underwent an initial telephone screening procedure to assess their eligibility for the study. Inclusion criteria was being between 50 and 80 years of age, residing at home, and if in the patient group, having a clinical diagnosis for AD and a family carer (relative/spouse) that knows them well and is willing to assist in the study. The exclusion criteria was having a previous history of alcohol/substance abuse, diagnosis of a psychiatric condition or any other significant medical conditions that may be likely to affect participation in the study (head injury, vision loss, mobility issues), and for the patients, diagnosis of a comorbid neurological condition that is not related to AD.

Signed informed consent was obtained from all participants and if they lacked mental capacity to give informed consent, from their legal guardians (applicable to patients only) prior to undergoing the experimental protocol. Ethical approval for this study was provided by the Faculty of Medicine and Health Sciences Research Ethics Committee at the University of East Anglia (FMH2017/18–123) as well as the National Health Service Health Research Authority (project ID 205,788; 16/LO/1366). All experiments were performed in accordance with the relevant guidelines and regulations.

### Experimental protocol

All participants underwent an experimental protocol which consisted of a VR spatial navigation testing session and a community spatial navigation testing session.

The VR navigation testing session was held in a quiet testing room in the university campus for the controls, whilst for patients this was held at a quiet room in their own home. In this session, the background demographics of all participants (i.e., age, gender, level of education, and duration lived at address) were collected from their carers. The participants’ previous history of getting lost in the community was also collected from their carers, in the form of a binary yes/no answer to the question ‘Has your care recipient ever gotten lost in the community before?’. In addition, the participants completed the Mini-Addenbrooke’s Cognitive Examination (Mini-ACE), which is a sensitive, validated cognitive screening test for dementia^24^. Following the cognitive screening, participants were then tested on their spatial navigation abilities using two non-immersive VR navigation tests on an iPad – the Virtual Supermarket Test and Sea Hero Quest^25,26^. Following the VR testing, the community navigation testing session was held on a separate day for all participants, where they completed an outdoor Detour Navigation Test in their own neighbourhood. Both VR and community navigation tests are detailed below.

### VR navigation—virtual supermarket test

**Figure 1:**
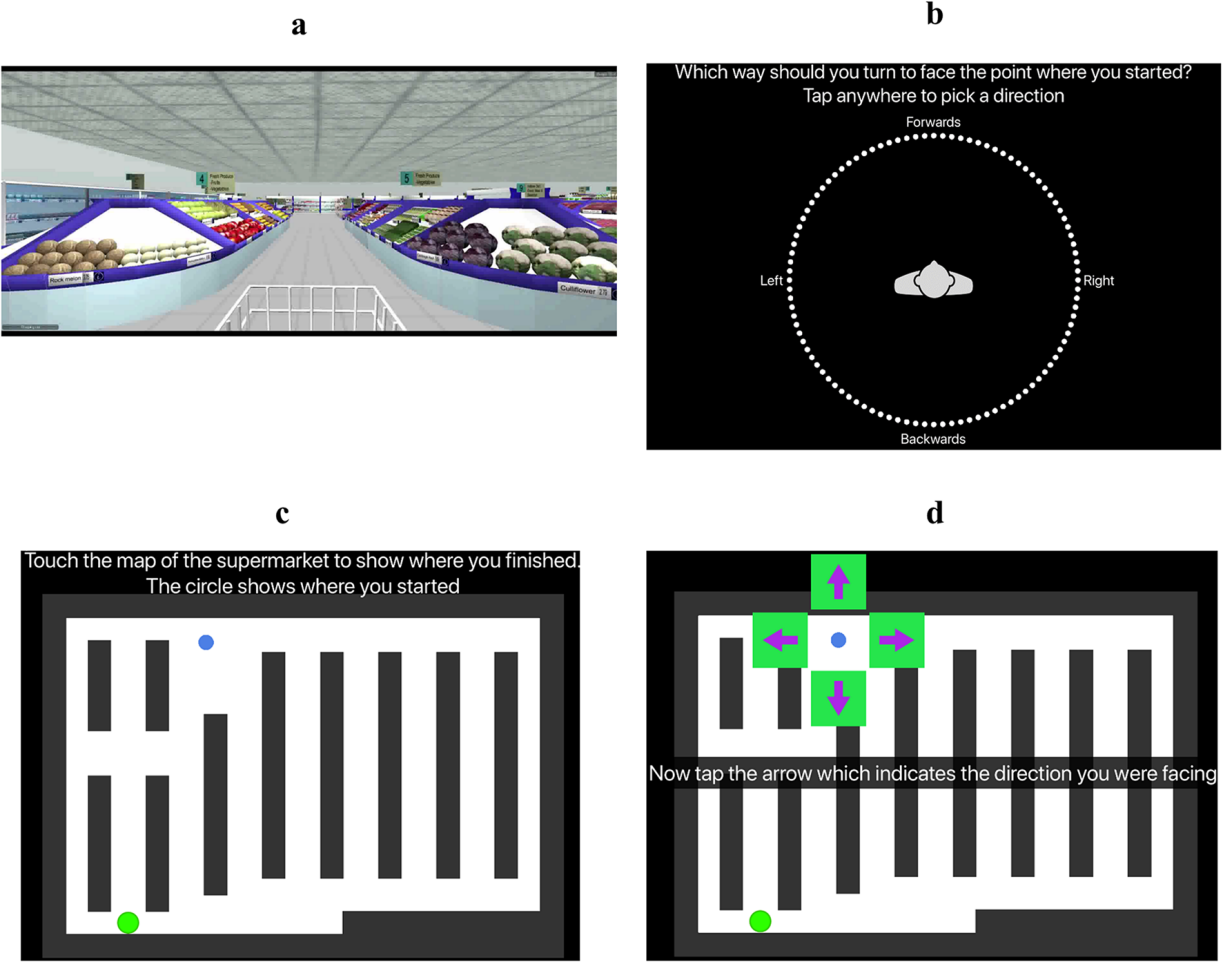
Illustration of the VST. (**a)** Participants are shown videos of a shopping trolley, from a first person perspective, moving along fixed routes in a supermarket, (**b**) Egocentric orientation component of task, where the direction of the starting location in relation to destination location must be indicated, (**c**) Allocentric orientation component of task, where the destination location must be indicated (blue circle represents example response) on a blank map of the supermarket with only the starting location labelled (green circle), (**d**) Heading direction component of task, where the direction faced when the trial finished must be indicated. Adapted from^59^.

The Virtual Supermarket Test (VST) is a spatial navigation test that assesses egocentric orientation, allocentric orientation, and heading direction. We chose this test as since it has been used by previous studies to highlight navigation impairments in AD patients, we wanted to explore if patient performance on this test relates to their spatial disorientation in the community^25,27,28^. In brief, an iPad is used to show participants 14 different videos (trials) lasting 20–40 s in duration, of a shopping trolley moving around a virtual supermarket, from a first person perspective (Fig. 1a). The virtual environment did not contain any salient landmarks or features, and is designed to test spatial navigation abilities without tapping into episodic memory, as any spatial representation acquired during testing is as a result of incidental encoding. In each video, participants begin at a fixed starting location and follow a different route, whilst making a series of 90° turns, to reach a specific destination in the supermarket (first 7 trials = 20 s, 3 turns; remaining 7 trials = 40 s; 5 turns). At the end of each trial, participants are asked three sets of questions to assess their egocentric orientation (Fig. 1b), allocentric orientation (Fig. 1c), and heading direction (Fig. 1d), respectively.

The VST has been described extensively in a previous study^25^. Further details of this test are also given in the supplementary material.

### VR navigation—sea hero quest

Sea Hero Quest (SHQ) is a mobile game that measures the spatial navigation abilities of individuals in laboratory and mass online settings. We chose this test as previous studies have shown its utility to assess navigation abilities in healthy individuals^26,29–31^, and we wanted to investigate whether the test can also identify navigation impairments in AD patients. Furthermore, navigation performance on this test has also been shown to relate to navigation performance in controlled RW environments^26^. Here, we take this one step further and assess if navigation performance on this test also relates to navigation performance in naturalistic RW environments that are highly personalised for each participant. The game involves players navigating a boat to various locations in a VR ocean environment on an iPad, and is composed of two types of levels—wayfinding and flare levels.

**Figure 2:**
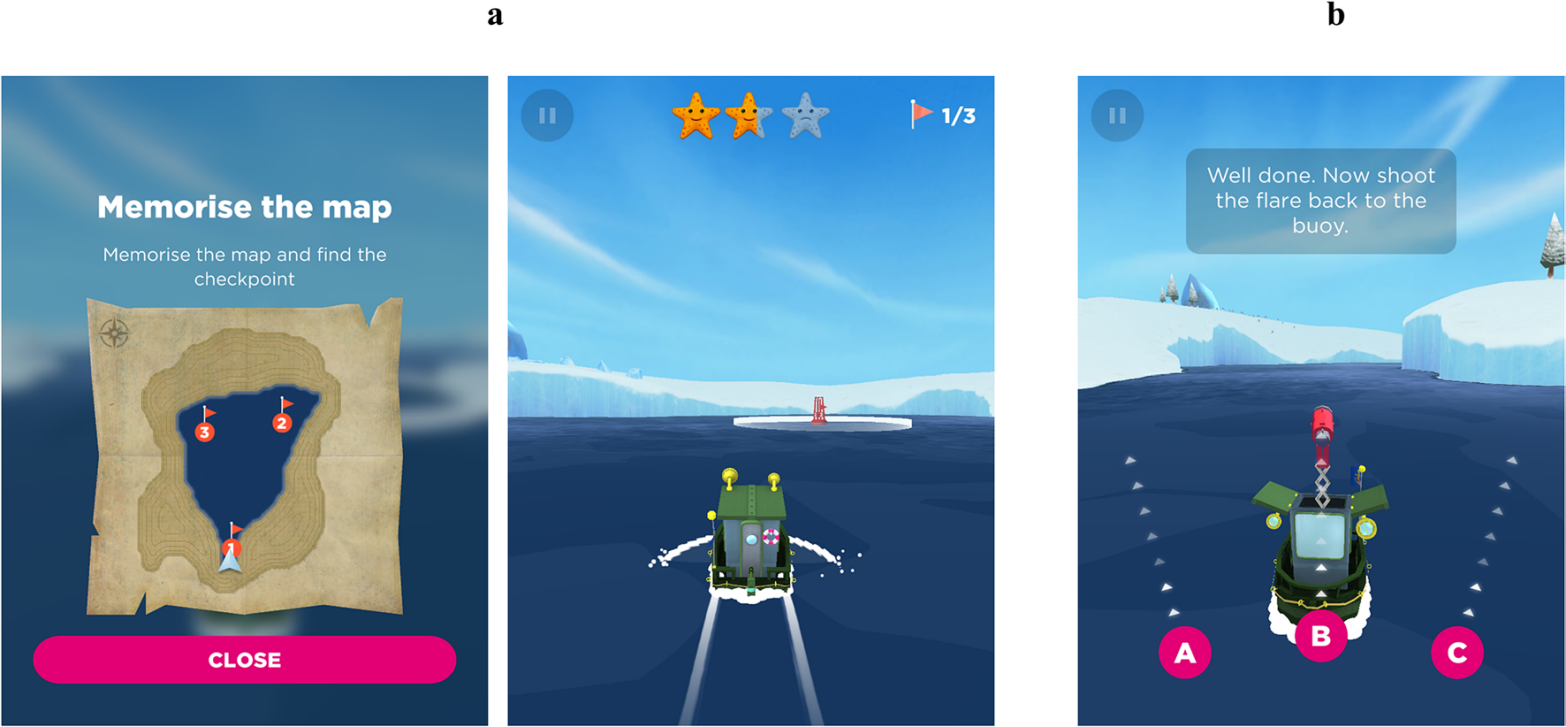
Illustration of SHQ. (**a**) Wayfinding level 6, where locations of three numbered checkpoints are first shown on a map. After the map disappears, participants have to navigate the boat to the numbered checkpoints in order, (**b**) Flare level 9, where participants navigate the boat from a starting location along the river, until they find a flare gun. Once found, the boat rotates by 180° clockwise and the participants are asked to shoot the gun in the direction of the starting location. Adapted from^59^.

In the wayfinding levels, players are first shown a map containing the start location and location of numbered checkpoints. They are instructed to study the map for as long as they need, and once they are ready, they tap on the screen and the map disappears. Their task is to then navigate the boat (from a first person perspective) to the checkpoints in order using their memory of the map (Fig. 2a). These levels necessitate participants to form and utilise a cognitive map for their wayfinding, and requires them to use more of an allocentric as opposed to an egocentric navigation strategy. The two outcome variables for the wayfinding levels are total distance travelled to visit all the checkpoints and total duration to complete the level. In our analysis however, we considered wayfinding distance as representing more the participants’ navigation ability compared to duration, and therefore we used this as our primary measure for these levels (see supplementary material for details). In this study, the wayfinding levels 6, 8, and 11 (which increases in complexity) were used, as these levels have been shown to challenge the navigation abilities of participants by a previous study^26^. Each of these levels had three checkpoints. However, with many of the AD patients finding level 6 (i.e., the relatively easiest level) quite challenging, the remaining levels were not administered for them; hence for the entire participant cohort, we used only wayfinding performance on level 6 for our analysis.

In the flare levels, participants are not provided with a map and are simply asked to navigate the boat from their starting location along various bend/turns on a river, until they find a flare gun. Once the flare gun is found, the boat rotates by 180° clockwise and the participants are asked to shoot the gun in the direction of where they think the starting location is, and are given three directions to choose from (*right, centre, left*) (Fig. 2b). Based on their response, participants are awarded one, two, or three stars for their flare accuracy, with higher stars indicating higher accuracy. Similar to the VST, this level requires participants to encode the starting location in relation to their current position, and hence measures their egocentric orientation. In line with a previous study^26^, the flare levels 9 and 14 were used and in addition, level 19 was also used. These three levels had only one 90° turn along the route. In order to challenge the participants to further identify those with better egocentric orientation, a final challenging level (i.e., level 49) was administered which had four 90° turns along the route. Flare accuracy for each level was weighted for the total number of turns in that level, and the total flare accuracy across all levels was the outcome measure.

SHQ has been described extensively in a previous study^26,30^. Further details of this test are also given in the supplementary material.

### Community navigation—detour navigation test

The Detour Navigation Test (DNT) is a novel real-world test that we are using for the first time, which tests the spatial navigation abilities of participants on an accompanied walk in a naturalistic community setting, that is also a highly familiar environment (i.e., their own neighbourhoods). We chose to use participants’ own neighbourhoods as the test setting to accurately simulate the most common RW situation where AD patients get lost in the community (i.e., during routine neighbourhood walks). An additional advantage of using a neighbourhood setting is that it enables us to overcome confounds of differences in spatial learning between controls and patients that would impact test performance if navigation was assessed in an unfamiliar environment^32^.

**Figure 3:**
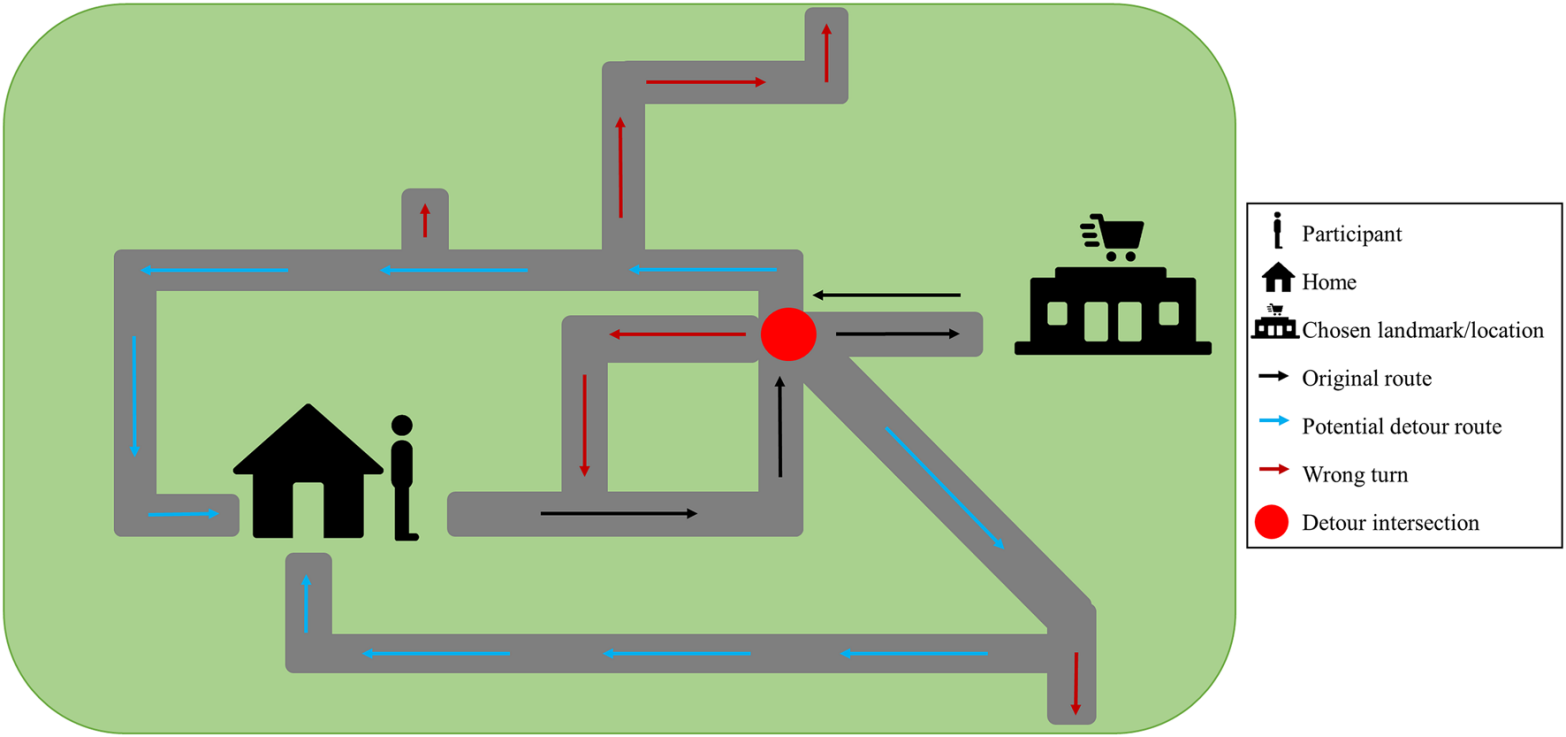
Illustration of the DNT. Participants navigate to a chosen landmark/location in their neighbourhood that they commonly visit using their usual route (i.e., original route); this route entails participants to predominantly use an egocentric navigation strategy. Once at the destination, participants are asked to navigate back home using the same route. Unknown to them, at the first intersection on the way back, they are asked to find an alternative route back home which does not overlap with the original route (i.e., detour route); this requires participants to utilise their cognitive maps of their neighbourhood to navigate, and hence entails the use of an allocentric navigation strategy. Adapted from^59^. Icons used in the figure – “Person” by Irene Hoffman, “Home” by Tauficon, “Supermarket” by Adrien Coquet, all from thenounproject.com.

At the end of the VR navigation testing session, the participants are asked to choose and describe a familiar route (Route A) from their house to a landmark/location in their neighbourhood that they often visit by foot, and this route is then marked by the experimenter on Google Maps. On a separate day, the participants are visited at home and accompanied by the experimenter on Route A. As taking familiar routes often lends itself more towards the use of egocentric navigation^33^, this route enables us to assess the use of this strategy during navigation in the community. Once at the end of Route A, the participants are instructed to navigate back to their house using the same route. Unknown to the participant, at the first intersection on the way back, they are asked to stop and find an alternative, detour route (Route B) back home that does not overlap at all (or if this is not possible, a route that overlaps as minimal as possible) with Route A. This task requires participants to use their cognitive maps of their neighbourhoods, and lends itself more towards the use of an allocentric navigation strategy. An overview of the DNT is illustrated in Fig. 3.

In this task, we measured spatial disorientation exhibited by the participants along these routes. Specifically, spatial disorientation is measured as the number of—(a) wrong turns made and (b) moments of hesitation. A wrong turn is defined as movement at an intersection (either straight or right/left turns) onto a path that is not marked as a viable alternative route on the map or onto part of the original route. For the latter, exceptions are made where the participant has no other alternative but to use part of the original route to get back home, in which case this is considered as an acceptable overlap and hence not marked as a wrong turn (eg. home located at end of a cul-de-sac). Participants could make a total of two consecutive wrong turns, at which point they would be brought back to the location before the first wrong turn was made and encouraged to try again. For the second variable, a moment of hesitation was defined as the participant either slowing down/stopping and looking around to aid orientation or verbally admitting that they are unsure about their whereabouts, in line with a previous study^21^.

Overall, for each participant the total number of wrong turns made and moments of hesitation were summed for both original/detour routes, and normalised for the respective total route distance and total route intersection number to give a route disorientation score. Furthermore, for patients, a composite disorientation score was calculated using the formula below:

$$\mathrm{Composite\, Disorientation\, Score}= \frac{\mathrm{Detour\, Route\, Disorientation\, Score}+1}{\mathrm{Original\, Route\, Disorientation\, Score}+1} .$$

Here, a constant of 1 was added to both original/detour route disorientation scores to overcome the division by zero problem, in cases where a patient exhibited no original route disorientation (i.e., score = 0). Hence, a composite disorientation score of 1 indicates that the patient had no disorientation in either the original or detour routes, a score greater than 1 indicates more disorientation on the detour than the original route, and a score less than 1 indicates more disorientation on the original than detour route.

Details on the individual routes (i.e., original and detour) that all participants took, with respect to total route distance and total route intersection number, are provided in the supplementary material.

### Data analysis

The data analysis was conducted in four different steps using RStudio software package version 3.4.2 (https://www.R-project.org/)^34^. In the first step, we investigated group differences in VR navigation by comparing patient performance on the VST (egocentric orientation, allocentric orientation, and heading direction) and SHQ (wayfinding and flare levels) to that of controls. In the second step, we assessed group differences in community navigation by comparing patient performance on the DNT (original and detour route disorientation scores) to that of controls. To assess group differences in the VR and community navigation variables, t-tests and/or Wilcoxon Rank Sum tests were used depending upon whether the variables had a normal/non-normal distribution.

In the third analysis step, we related patient performance on the VR navigation tasks to that of their community navigation. Here, we investigated whether any of the VR navigation variables (i.e., VST and SHQ variables) predict patients’ composite disorientation score on the DNT using linear regression models. In the fourth and final analysis step, we explored whether patient performance on any of the VR navigation variables predict whether they are at a high risk for spatial disorientation in the community. For this, we first divide the patients into high/low risk groups based on their composite disorientation score on the DNT. We then select the VR navigation variables that significantly predicted DNT composite disorientation score from step three, and assess how well these variables predict risk classification using binomial logistic regression models. Steps three and four were not conducted for the controls as we do not expect controls to experience disorientation in their own neighbourhoods, and hence are not interested in relating this outcome measure with their VR navigation performance.

## Results

### Participant demographics

When comparing the patients and controls on the demographic variables, no significant group differences were seen in age (W = 149.5, *p* = 0.440), gender (X^2^ = 0.005, *p* = 0.939), or duration that the participants lived at their address (W = 198, *p* = 0.699). Controls were significantly more educated (W = 285, *p* = 0.002, d = 1.12) and had a higher Mini-ACE score than the patients (W = 351.5, *p* < 0.001, d = 2.79); the scores of all patients on this test met the upper cut-off of ≤ 25/30, indicating the likely presence of dementia. The majority of the patients (n = 12) were reported as having had a prior history of at least one getting lost episode in the community by their carers (Table 1).

**Table 1:** Participant Demographics.

Variable	Controls (Mean; SD)	AD Patients (Mean; SD)	Significance (p-value)	Effect Sizes (Cohen’s d)
Sample Size	21	16		–
Age	68.36 (7.57)	70.25 (6.63)	ns	–
Gender (Males, Females)	10 M, 13F	8 M, 8F	ns	–
Education (Years)	15.65 (2.96)	12.81 (1.72)	**	1.12
Duration Lived at Address (Years)	15.04 (11.27)	15.85 (16.33)	ns	–
Prior Getting Lost History	–	12	–	–
Mini-ACE Score	28.59 (1.43)	18.25 (5.47)	***	2.79

**p < 0.01, ***p < 0.001, ns = not significant.

### Differences in VR navigation

Our results for the VST showed that patients had significantly worse performance on the egocentric orientation (W = 329, *p* < 0.001, d = 2.47), allocentric orientation (t =  − 3.107, *p* = 0.004, d = 1.04), and allocentric heading direction (W = 334.5, *p* < 0.001, d = 2.57) components when compared to the controls.

Our results for the egocentric flare levels on SHQ showed that patients had no significant differences in their weighted flare accuracy scores when compared to controls (W = 199, *p* = 0.297). However, patients had worse performance than controls on the allocentric wayfinding level 6 as they had a significantly higher distance travelled (W = 59, *p* = 0.0015, d = 1.24) and duration taken to complete the level (W = 77, *p* = 0.011, d = 1.13). As map view duration can influence performance on the wayfinding levels, we ran one-way ANCOVAs to see whether these effects remained even after controlling for this covariate. As the distance travelled and duration variables had a non-normal distribution, we inverse transformed these variables to alleviate the positive skewness, which enabled us to run this parametric test. Our results for the ANCOVAs show that these effects remained even after controlling for map view duration (F(1,33) = 5.828, *p* = 0.021 for distance travelled and F(1,33) = 6.599, *p* = 0.014 for duration to complete level).

Results of group differences for all VR navigation variables are summarised in Table 2.

**Table 2:** Overview of Group Differences in VR/Community Navigation Variables.

Navigation Test	Variable	Controls (Mean; SD)	Patients (Mean; SD)	Significance (p-value)	Effect Size (Cohen’s d)
VST (VR)	Egocentric Orientation (% Correct)	81.49 (21.67)	30.35 (19.25)	***	2.47
	Allocentric Map Orientation (Displacement; % of Map Size)	18.57 (7.16)	26.44 (8.07)	**	1.04
	Heading Direction (% Correct)	83.76 (16.37)	34.37 (22.46)	***	2.57
SHQ (VR)	Wayfinding Distance Score	0.71 (0.27)	1.21 (0.55)	**	1.24
	Wayfinding Duration Score	0.73 (0.27)	1.25 (0.63)	*	1.13
	Flare Accuracy Score	2.30 (0.54)	2.12 (0.54)	ns	-
DNT (Community)	Original Route Number of Wrong Turns	0.00 (0.00)	0.00 (0.00)	-	-
	Original Route Moments of Hesitation	0.00 (0.00)	0.01 (0.07)	-	-
	Original Route Disorientation Score	0.00 (0.00)	0.01 (0.07)	ns	-
	Detour Route Number of Wrong Turns	0.00 (0.00)	0.02 (0.09)	-	-
	Detour Route Moments of Hesitation	0.001 (0.008)	0.23 (0.47)	-	-
	Detour Route Disorientation Score	0.001 (0.008)	0.25 (0.50)	**	0.76

*p < 0.05, **p < 0.01, ***p < 0.001, ns = not significant.

### Differences in community navigation

All participants were able to successfully complete the DNT, except for one patient who could not complete the detour route due to physical mobility issues. Data for this participant was not included for any of the downstream analyses. Only one patient made two consecutive wrong turns (on the DNT) and hence required assistance from the experimenter to bring them back to the location before the first wrong turn was made.

Our results for the DNT showed that there were no significant differences between patients and controls for their original route disorientation scores (W = 147, *p* = 0.259), however patients had a significantly higher disorientation score when compared to controls for the detour route (W = 99, *p* = 0.007, d = 0.76) (Table 2).

### Prediction of community navigation from VR navigation—linear regression

The results of our linear regression models showed that for the VST, neither patient performance on egocentric orientation (β =  − 0.0006, *p* = 0.931, R^2^ = 0.0005), allocentric orientation (β = 0.018, p = 0.302, R^2^ = 0.081), nor heading direction (β =  − 0.005, p = 0.342, R^2^ = 0.069) significantly predicted their composite disorientation score on the DNT.

**Figure 4:**
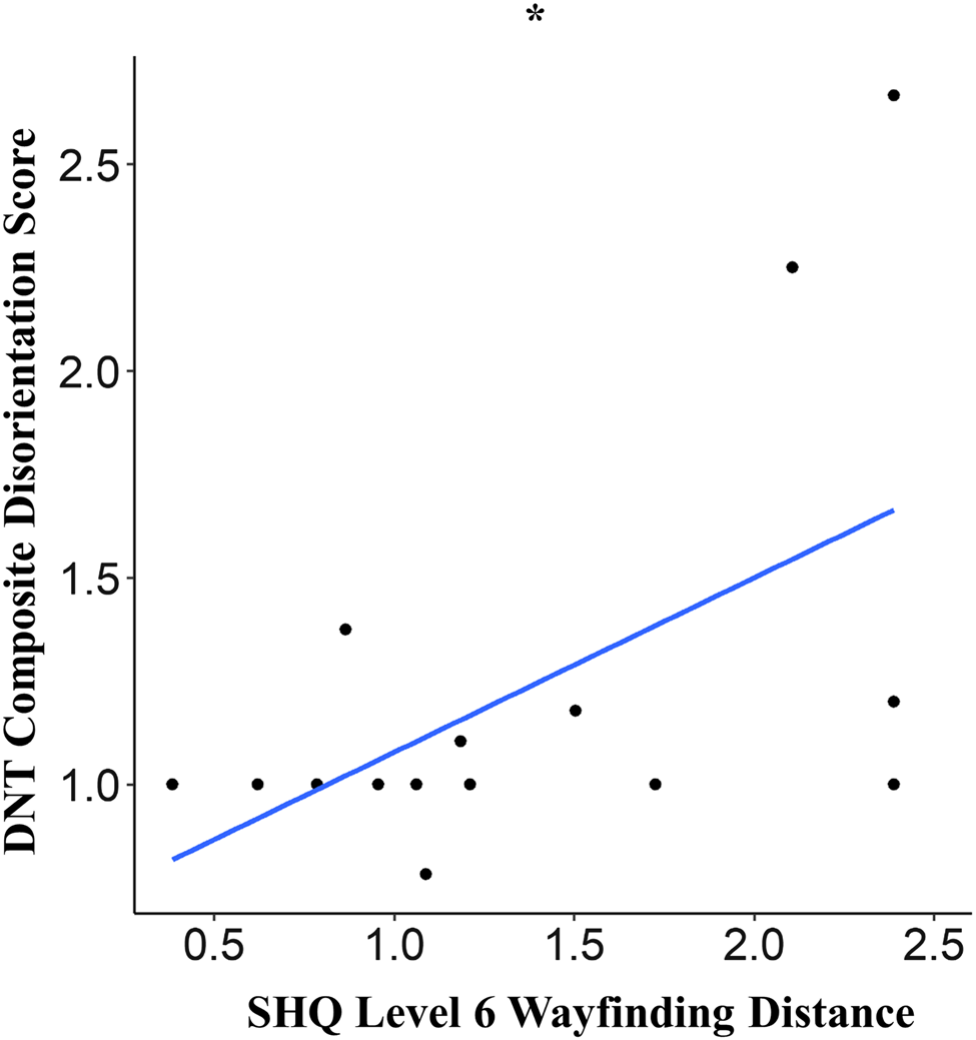
Linear regression model. Patient performance on SHQ level 6 wayfinding distance significantly predicted their DNT composite disorientation score (*p* = 0.034, R^2^ = 0.29). Adapted from^59^.

For SHQ, we found that two patients struggled quite extensively on the practice wayfinding levels, and hence level 6 was not administered for them. Based on this, we assume that if this level had been administered, both patients would have had performed more poorly on the wayfinding variables when compared to the other patients. Hence to include these patients in our regression models and increase its statistical power, we assign them both predicted scores for wayfinding distance and duration, which were the scores of the patients who performed most poorly on these variables on this level. Subsequently, our results showed that both higher wayfinding distance and duration on level 6 significantly predicted increased composite disorientation score on the DNT (β = 0.422, p = 0.034, R^2^ = 0.29, f^2^ = 0.42 and β = 0.357, p = 0.046, R^2^ = 0.27, f^2^ = 0.37 respectively). Importantly, both models had normally distributed residuals. With distance travelled being our primary measure of the navigation ability on the wayfinding levels, we consider the model with this variable as the predictor as our main model (Fig. 4). The figure showing the significant relationship between SHQ wayfinding duration and DNT composite disorientation score is provided in the supplementary material.

As a post-hoc analysis, we were interested to see if the significant relationships between SHQ level 6 wayfinding distance and duration with DNT composite disorientation score remained after removing the two patients (mentioned above) who did not complete this level. After removing these patients, our results showed that there were statistical trends for higher wayfinding distance and duration on SHQ level 6 predicting increased composite disorientation score on the DNT for the patients (β = 0.322 , p = 0.071, R^2^ = 0.26, f^2^ = 0.36 and β = 0.259, p = 0.098, R^2^ = 0.22, f^2^ = 0.29 respectively).

### Prediction of community navigation from VR navigation—logistic regression

To identify whether SHQ wayfinding distance performance of the patients can predict risk for spatial disorientation in the community, we divided the patients into two risk groups. Patients who exhibited disorientation on the DNT (i.e., composite disorientation score ≠ 1; n = 7) were classified as high risk for spatial disorientation, whilst the rest (i.e., composite disorientation score = 1; n = 8) were classified as low risk. A binomial logistic regression was then run to see how well SHQ wayfinding distance performance predicts patients’ group membership. The results of this regression showed that SHQ wayfinding distance performance could not significantly predict patients at a high risk for RW spatial disorientation (OR = 3.70, *p* = 0.155).

As a post-hoc analysis, we were also interested to see if SHQ wayfinding duration performance (which also significantly related to DNT composite disorientation score) could predict patients’ risk classification, and ran a second binomial logistic regression. The results of this model showed that SHQ wayfinding duration performance could also not significantly predict patients at a high risk for RW spatial disorientation (OR = 2.35, *p* = 0.259).

Lastly, as an exploratory analysis, we were interested to see if any of the measures on the VST and SHQ could predict the experience of spatial disorientation in the community across all our study participants (regardless of whether a patient or control). Details of this analysis are provided in the supplementary material.

## Discussion

From a VR perspective, in line with our hypothesis, we found that AD patients exhibited impairments in all aspects of the VST when compared to the controls. Meanwhile on SHQ, we found that patients only exhibited impairments on the wayfinding (i.e., allocentric) levels and not the flare (i.e., egocentric) levels. From a community perspective, contradictory to the hypothesis, we found that patients’ performance on their original route (i.e., where a predominantly egocentric strategy is most likely used) was comparable to controls. In line with the hypothesis however, our results showed that the patients performed significantly worse than controls on their detour route (i.e., where a predominantly allocentric strategy is most likely used). When relating the VR and community navigation variables for the patients, we found that only performance on SHQ level 6 wayfinding distance related to performance on the DNT. Despite this, SHQ level 6 wayfinding distance performance did not predict patients at a high risk for spatial disorientation, where risk was classified based on DNT performance.

In more detail, our findings of patients being impaired on all components of the VST is in agreement with previous studies^25,28^. Regarding our findings from SHQ, which was used for the first time here to assess navigation in AD patients, it was quite surprising that the patients performed similar to controls on the flare levels of this test, considering that these levels measure egocentric orientation in a similar way to the VST. This null result could potentially be explained by the flare levels being relatively easier, having on average relatively fewer turns along the route and fewer multiple choice answer options when compared to the egocentric component of the VST. This considered, our results suggest that the flare levels of SHQ, at least using 3 levels and the ones used in this study, lack sensitivity to detect egocentric orientation impairments in AD patients. Overall, our findings from the VST and SHQ add to the existing literature on AD patients experiencing spatial disorientation in VR environments^14^.

Regarding our findings from the DNT, to the best of our knowledge, this is the first study to systematically assess the ability of AD patients to use egocentric and allocentric strategies for navigation in a familiar community setting. Findings from previous real world navigation studies in AD suggest that patients are impaired in using both egocentric and allocentric navigation strategies in controlled, unfamiliar environments^35–39^. We extend these findings by showing that in a naturalistic, familiar environment, patients exhibit impairments specifically when it may be beneficial to use an allocentric strategy to solve a navigation task as opposed to an egocentric strategy. It is interesting to note that despite these impairments, all patients (except one) were able to successfully complete the task (i.e., use an alternative route to find their way back home) without getting lost or requiring external assistance. This finding is consistent with previous research showing that AD patients were able to use their long-term knowledge of a familiar environment to successfully navigate in a VR simulation of that environment^40^. Our results also parallel observations of successful navigation of familiar environments by patients^41–43^ and rats^44^ with damage to their hippocampus. It is possible that such navigation draws on the caudate nucleus, which has been shown in recent work to play a more prominent role in flexible goal-directed navigation than previously thought^45,46^.

The differential impairment for patients on the detour route may be explained by the extra demands on the extended hippocampal network required by detours^47^. In environments where future alternative routes can be considered for navigation, hippocampal place cells have been shown to be ‘replay’ future paths to goals^48–50^. Such replay is disrupted in AD mouse models^51^ and thus, it is possible that disrupted replay in AD patients leads to impaired navigation when detours are required in the DNT. However, future studies are required to further elucidate the relationship between impairments at the cellular level and the navigation impairments observed for AD patients in the community.

When relating patient performance on the VR navigation tasks to that of the DNT, we found that worse SHQ level 6 wayfinding performance significantly predicted increased DNT composite disorientation score. This finding was seen after including the two patients who did not complete this level (by assigning them predicted scores); although in our post-hoc analysis we observed that removing these patients resulted in this significant finding reducing into a statistical trend, the effect sizes for our post-hoc model (f^2^ = 0.36) still indicate a large effect for the relationship between both variables^52^. This is not surprising as both tasks are quite similar in nature, requiring participants to take a novel path to a specific goal. Further, our findings are supported by results from a previous study which showed that SHQ wayfinding performance correlated with wayfinding performance in naturalistic, real world city environments (London and Paris) for healthy participants^29^. Taken together with results from this previous study, our finding highlights the real-world application of the wayfinding levels on SHQ in predicting spatial disorientation for patients in situations where it is beneficial to use cognitive maps for navigation in the community. However, with this finding being based on a limited sample of AD patients, validation using a relatively larger sample size is warranted.

In contrast to results from SHQ, we found that patients’ impairments on the VST (i.e., egocentric, allocentric, and heading direction components) did not relate to their performance on the DNT. The reason for this null result is at present unclear, however it could potentially be due to differences in how the different aspects of navigation were measured in both tasks. Specifically, the DNT does not explicitly measure patients’ heading direction or patients’ allocentric knowledge of their destination’s location on a blank map as the VST does, and hence could explain why these variables did not relate to the DNT composite disorientation score. Furthermore, although the use of an egocentric navigation strategy is measured in both tasks, differences exist in the way it is measured. In the VST, this is measured by looking at the ability of patients to correctly point to the starting location after passively navigating through a route. However in the DNT, egocentric navigation strategy use is measured mainly by looking at the ability of patients to correctly use a well familiar route to actively navigate to a destination (i.e., original route); although not explicitly measured, patients are likely using this strategy on this task either by using visible landmarks or their sequential knowledge of left–right turns that need to be made to inform their navigation decisions^53^. Indeed, such differences in how egocentric navigation strategy use were measured in both tasks could explain why patient scores on the two tests did not relate to one another.

We found that patient performance on none of the VR tests, but especially on the egocentric orientation component of the VST, predicted their risk for spatial disorientation in the community. This null result could be due to differences in how navigation is carried out in both types of environments. Specifically, the VR tests are designed to segregate as much as possible the use of both egocentric and allocentric navigation strategies from each other whereas in the real-world, these strategies are often used in combination with one another when one navigates^54^, with the relative weighting given to each type of strategy differing according to task condition (i.e., original route – egocentric > allocentric; detour route – allocentric > egocentric). Almost all (except one patient) in the high risk for spatial disorientation group experienced disorientation on the detour route of the DNT, where they are required to predominantly use an allocentric navigation strategy. In light of findings from previous studies that patients may use an egocentric strategy to aid their navigation and compensate for deficits in using an allocentric navigation strategy, we speculate that this disorientation could have resulted due to the inability of these patients to effectively do so. The fact that the VR tests used do not in design measure such interaction between both types of navigation strategies could be an underlying reason as to why performance on these tests did not predict risk for spatial disorientation in the community. Another reason for the inability of the VR tests to predict risk for spatial disorientation in the community could be due to differences in the level of disease progression for patients. Specifically, it has previously been reported that for AD patients, preference for the use of an egocentric strategy to potentially compensate for allocentric deficits increases with disease severity^17^. As patients with relatively higher disease severity may be more likely to use an egocentric strategy to compensate for their allocentric deficits on the detour route, it could be that a relationship between egocentric orientation abilities in the VR tests and disorientation on the DNT might only be seen when considering just these individuals. Since we did not measure level of disease severity for our patients, it is at present unclear to what extent inter-individual differences on this variable existed in our sample. Lastly, our current sample sizes (n = 8 low risk for disorientation; n = 7 high risk for disorientation) do not meet the widely adopted 10 events per variable guideline for binary logistic regression analysis^55^, and this factor could have potentially underlined the null results seen in our analysis. Indeed, the significant results we obtained from the exploratory logistic regression analyses using all our study participants (see Supplementary Material) suggests that our main analysis focused on patients alone was underpowered, and having a larger sample size for this could potentially show an ability for the VR tests to predict risk classification. Hence overall, our current findings do not provide sufficient insight to validate our hypothesis that AD patients who are poor egocentric navigators are the ones that exhibit a high risk for spatial disorientation in the community.

To further elucidate our hypothesis, it would be beneficial for future studies to employ a VR task that systematically measures participants abilities to use an egocentric navigation strategy to aid their navigation under an allocentric condition (i.e., where they are expected to experience disorientation), similar to a paradigm used by a previous study^56^. Further, the extent to which patients are actually using an egocentric strategy to aid their navigation on the DNT detour route should also be determined by taking into account their level of disease severity and asking them to elaborate on the navigation strategies that they used for this route. It should then be explored whether those that experienced more disorientation on the DNT are weaker in their ability to use an egocentric navigation strategy for reorientation as identified by the VR task.

Despite our novel findings in this study, there are some limitations that need to be mentioned. Firstly, we did not control for participants’ familiarity of the detour route on the DNT. Although the detour route is designed to elicit participants to predominantly use an allocentric strategy to navigate, familiarity with the detour route can influence the extent to which this strategy is actually used. Specifically, participants that are very familiar with the detour route would be expected to have been mainly using an egocentric strategy to navigate, with only limited involvement of an allocentric strategy, whereas those that are relatively less familiar with the detour route would be expected to be using more of an allocentric strategy (as intended). Therefore, the extent to which inter-individual differences in familiarity with the detour route explain why some patients experienced disorientation on this route and others did not is at present unclear. Future studies could attempt to use sensor-based GPS measurements of patients’ outdoor movements in the community under free-living conditions to provide an objective measure of how familiar the participants are with the detour route. Another limitation pertains to our methodology of hesitation behaviour on the DNT being measured visually by a single experimenter, with no time limits thresholds imposed to identify this behaviour. This approach has the potential limitations of being subjected to bias and not capturing more subtle moments of hesitation, which may have gone unnoticed. A recent study has shown that hesitation behaviour of AD patients can be measured objectively by looking at the spatio-temporal gait patterns (i.e., step patterns) of their walking paths in controlled real world environments using inertial measurement units^57^. Further, results from another study showed that disorientation behaviour could be identified from accelerometer data of patients navigating through a naturalistic real world city environment^58^. These studies suggest that a gait-based approach is indeed feasible to objectively identify spatial disorientation behaviour, and future studies should explore the possibility of using sensor devices, and imposed time limit thresholds, to more accurately measure this behaviour from patients’ outdoor navigation in the community.

In conclusion, our results showed that spatial navigation impairments can be detected in AD patients using VR navigation tests as well as when performing goal-oriented navigation tasks in a familiar, community environment. However, the VR navigation tests in general were not able to predict which patients are at a high risk for spatial disorientation in the community. As far as we are aware, this is the first study to relate patient navigation performance in VR environments to their risk for spatial disorientation in the community. An important direction for future studies would be to explore and identify VR navigation tests and measures that are effective in predicting patients’ risk for spatial disorientation in the community. Doing so can have important clinical implications, as patient navigation performance on these tests can potentially be used to inform the clinicians of their risk for spatial disorientation as well as the implementation of interventions to prevent these episodes from actually occurring for patients in the community.

## Supplementary Information

Supplementary Information.

## Data Availability

The data used in this study is available from the authors upon reasonable request.
