# Supplementary material for: Predicting real world spatial disorientation in Alzheimer’s disease patients using virtual reality navigation tests
Source: Sci Rep. 2022 Aug 4;12:13397. doi: 10.1038/s41598-022-17634-w (PMC9352716; doi:10.1038/s41598-022-17634-w)
Supplement: Supplementary file 1 — Supplementary Information. [file 41598_2022_17634_MOESM1_ESM.docx]

**SUPPLEMENTARY MATERIAL**

PARTICIPANT RECRUITMENT

The majority of patients were recruited from community memory clinics, whereby patients are referred by clinicians from this clinic to participate in dementia research. Some patients were also recruited from the Join Dementia Research website (<https://www.joindementiaresearch.nihr.ac.uk/>), which is an online service allowing individuals with dementia, their carers, as well as healthy individuals to self-register and volunteer to participate in dementia research studies. Lastly, few patients were also recruited from memory and dementia cafes/fayres held by our study team in the community.

The majority of the healthy controls recruited comprised of individuals who had participated in other studies run by our team and who had indicated an interest to participate in future research. In addition, individuals who expressed their interest to volunteer in the study as a result of word of mouth publicity were also recruited as controls for the study.

## VIRTUAL SUPERMARKET TEST

To assess egocentric orientation, participants are asked to indicate the direction of the starting location in relation to their current location (i.e., destination). Here, participants are instructed to give two directional components for their response (i.e., *front left, back right, front right, etc.*) (Fig. 1b). For each trial, a response was scored as being correct only if both directional components were given correctly, and the outcome measure was total percentage of correct answers across all trials.

Participants are next assessed on their allocentric orientation, where they are shown a blank map of the supermarket with only the starting location labelled, and are instructed to mark on the map where they think the destination is (Fig. 1c). Here, the outcome measure is the distance error (i.e., displacement) between the participant’s response and correct location, and this was measured and expressed as percentage of map size.

Lastly, on the map of the supermarket the participants are asked to indicate their heading direction (i.e., the direction that they were facing when the trial finished). They could give their response in terms of the four cardinal directions (*north, east, south, and west*) (Fig. 1d). Similar to egocentric orientation, the outcome measure here was the total percentage of correct answers across all trials.

## SEA HERO QUEST

On the wayfinding levels, participants are timed as they complete the level; if they exceed a set time threshold, an arrow appears that points in the Euclidean direction of the goal location in order to aid their wayfinding. This arrow appeared in 13.04% of the controls and in 64.28% of the patients who completed the wayfinding level 6. A Fisher’s Exact Test showed that there were significant group differences in the likelihood of the arrow appearing (p = 0.002), with the arrow being more likely to appear for the patients when compared to the controls.

The two outcome variables for the wayfinding levels are total distance travelled to visit all the checkpoints and total duration to complete the level. Here, higher distance travelled and duration to complete the level are considered to reflect less efficient navigation and hence, worse wayfinding performance. Here, a caveat for increased wayfinding duration is that it can also reflect participant’s use (or lack of) of the boat’s acceleration (i.e., swiping up on the iPad screen temporarily increases the boat’s speed) and hence can be indicative of more non-navigational factors like navigation confidence or personal preference of boat’s speed. Hence, we consider wayfinding distance as representing more the participants’ navigation ability compared to duration, and use this as our primary measure for these levels.

Importantly, to account for inter-individual differences in gaming proficiency, two practice levels are administered at the start of the game, where participants memorise and navigate to the location of a single checkpoint. In these levels the checkpoint is simply located at the end of a straight path, and hence these levels do not require much spatial navigation ability and instead measure gaming proficiency. Each participant’s score on the wayfinding levels were then normalised for the sum of their scores on the practice levels, to account for their gaming proficiency.

## DETOUR NAVIGATION TEST

## Details of the original and detour routes taken by all participants (controls & AD patients) in the DNT, with regards to total route distance and number of road intersections, are provided in the tables below.

## **Supplementary Table 1:** Details of Routes Taken by Controls in the DNT

| Control Participant ID | Original Route Distance (Miles) | Original Route Number of Road Intersections | Detour Route Distance (Miles) | Detour Route Number of Road Intersections |
| --- | --- | --- | --- | --- |
| 1 | 0.50 | 8 | 1.00 | 10 |
| 2 | 1.00 | 18 | 1.19 | 21 |
| 3 | 0.90 | 19 | 0.81 | 13 |
| 4 | 0.40 | 11 | 0.50 | 13 |
| 5 | 0.70 | 15 | 1.13 | 14 |
| 6 | 0.60 | 15 | 1.09 | 16 |
| 7 | 0.60 | 15 | 0.64 | 15 |
| 8 | 0.60 | 15 | 0.90 | 26 |
| 9 | 0.60 | 15 | 0.82 | 21 |
| 10 | 0.50 | 15 | 0.64 | 12 |
| 11 | 0.21 | 4 | 0.24 | 9 |
| 12 | 0.50 | 8 | 0.90 | 20 |
| 13 | 0.50 | 3 | 0.70 | 9 |
| 14 | 0.80 | 17 | 0.92 | 20 |
| 15 | 0.80 | 9 | 1.27 | 4 |
| 16 | 0.70 | 16 | 0.92 | 12 |
| 17 | 0.60 | 19 | 1.12 | 23 |
| 18 | 0.50 | 12 | 0.85 | 23 |
| 19 | 0.50 | 17 | 0.83 | 10 |
| 20 | 0.83 | 19 | 0.87 | 15 |
| 21 | 0.80 | 16 | 0.89 | 30 |

## **Supplementary Table 2:** Details of Routes Taken by Patients in the DNT

| Patient Participant ID | Original Route Distance (Miles) | Original Route Number of Road Intersections | Detour Route Distance (Miles) | Detour Route Number of Road Intersections |
| --- | --- | --- | --- | --- |
| 1 | 0.90 | 23 | 0.96 | 22 |
| 2 | 0.90 | 9 | 0.90 | 10 |
| 3 | 0.80 | 20 | 0.23 | 8 |
| 4 | 0.1 | 1 | 0.30 | 2 |
| 5 | 1.36 | 2 | 1.20 | 5 |
| 6 | 0.4 | 7 | 0.90 | 8 |
| 7 | 0.70 | 16 | 1.42 | 23 |
| 8 | 0.46 | 3 | 0.70 | 16 |
| 9 | 0.60 | 5 | 0.70 | 16 |
| 10 | 0.40 | 10 | 0.43 | 11 |
| 11 | 0.40 | 5 | 0.50 | 10 |
| 12 | 0.40 | 11 | 0.60 | 17 |
| 13 | 0.50 | 13 | 0.62 | 17 |
| 14 | 0.18 | 6 | 0.20 | 5 |
| 15 | 0.2 | 3 | 0.53 | 3 |

##
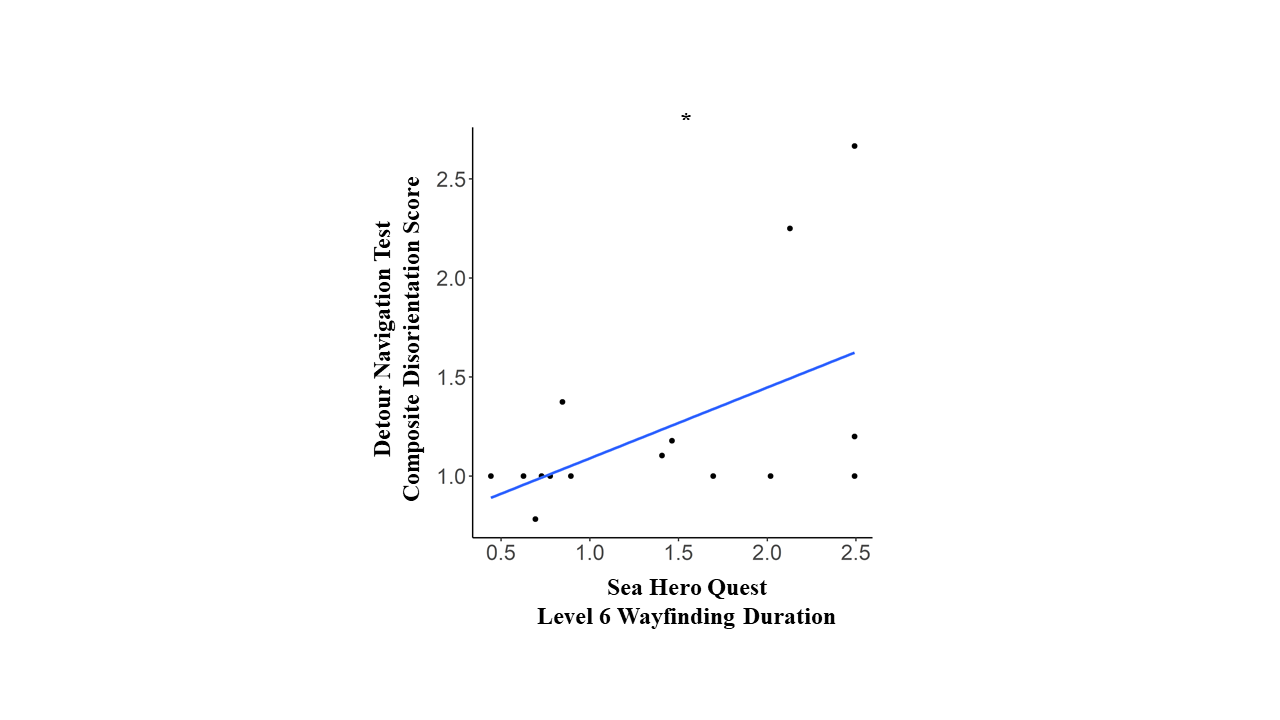
PREDICTION OF COMMUNITY NAVIGATION FROM VR NAVIGATION – LINEAR REGRESSION

**Supplementary Figure 1:** Linear regression model. Patient performance on SHQ level 6 wayfinding duration significantly predicted their DNT composite disorientation score (p = 0.046, R^2^ = 0.27).

## PREDICTION OF COMMUNITY NAVIGATION FROM VR NAVIGATION – LOGISTIC REGRESSION

For our final, exploratory analysis, we divided all our participants into two groups – those that experienced spatial disorientation in the DNT (composite disorientation score ≠ 1; n = 8) and those that did not (composite disorientation score = 1; n = 28). We then ran separate binomial logistic regressions, with each of the VST and SHQ measures as predictor variables respectively. The results showed that having higher VST egocentric orientation (OR = 0.96, p = 0.021) and heading direction (OR = 0.96, p = 0.032) scores significantly decreased odds of being in the disoriented group whilst having higher VST allocentric orientation (OR = 1.14, p = 0.027), SHQ wayfinding distance (OR = 6.75, p = 0.012) and wayfinding duration (OR = 4.52, p = 0.021) scores significantly increased odds of being in the disoriented group. Meanwhile, VST flare accuracy scores was not significantly associated with group membership (OR=1.82, p = 0.448). To assess and compare the relative group classification accuracy of these VR measures, separate receiver operating characteristic (ROC) curves were computed, and the area under curve (AUC) values were used. The results showed that all measures had similar AUC values (VST egocentric orientation = 0.781, VST allocentric orientation = 0.776, VST heading direction = 0.756, SHQ wayfinding distance = 0.799, and SHQ wayfinding duration = 0.750). A comparison of the ROC curves of the measures with the two highest AUC values is plotted below.


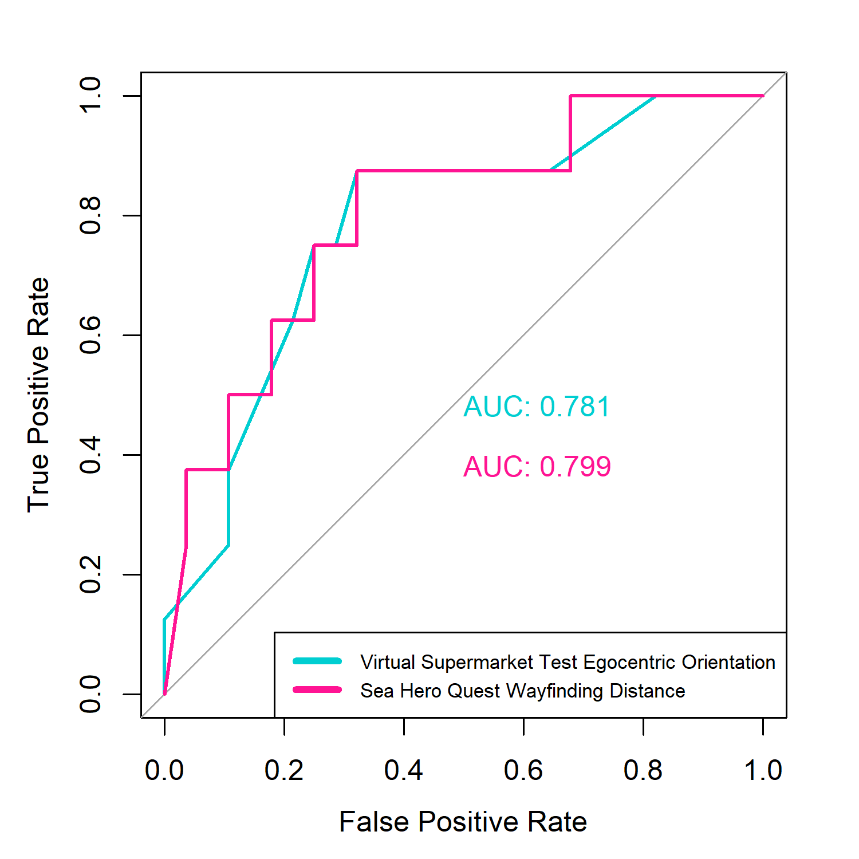


**Supplementary Figure 2:** ROC Curves showing group classification accuracies (i.e., AUC values) of the VST Egocentric Orientation and SHQ Wayfinding Distance measures of participants into the disoriented vs. not disoriented groups.
